# The interplay between excess weight and hyper-glycemia on NCDs in Italy: results from a cross-sectional study

**DOI:** 10.1007/s00592-024-02296-z

**Published:** 2024-05-13

**Authors:** Vincenzo Atella, Federico Belotti, Matilde Giaccherini, Gerardo Medea, Andrea Piano Mortari, Paolo Sbraccia, Antonio Nicolucci

**Affiliations:** 1 Department of Economics and Finance, University of Rome Tor Vergata, Rome, Italy; 2 CEIS Tor Vergata, University of Rome Tor Vergata, Rome, Italy; 3 Italian College of General Practitioners, Florence, Italy; 4 Department of Systems Medicine, University of Rome Tor Vergata, Rome, Italy; 5 Center for Outcomes Research and Clinical Epidemiology—CORESEARCH, Corso Umberto I, 103, 65122 Pescara, Italy

**Keywords:** Overweight, Obesity, Comorbidities, Glucose metabolism alterations, Epidemiology, Clinical burden

## Abstract

**Purpose:**

To evaluate the prevalence of chronic comorbidities according to BMI classes and assess the interplay between excess body weight and blood glucose abnormalities in increasing the risk of major chronic diseases.

**Methods:**

The study is based on data from the Health Search/IQVIA Health LPD Longitudinal Patient Database, an Italian general practice registry, with data obtained from electronic clinical records of 800 general practitioners throughout Italy. Data relative to the year 2018 were analyzed. The study population was classified according to BMI (normal weight, overweight, and obesity classes 1, 2 and 3) and glucose metabolism status (normoglycemia—NGT; impaired fasting glucose—IFG; diabetes mellitus—DM). Comorbidities were identified through ICD-9 CM codes.

**Results:**

Data relative to 991,917 adults were analyzed. The prevalence of overweight was 39.4%, while the prevalence of obesity was 11.1% (class 1: 7.9%, class 2: 2.3%, class 3: 0.9%). In the whole population, the prevalence of DM and IFG was 8.9% and 4.2%, respectively. Both overweight and obesity were associated with an increasing prevalence of glucose metabolism alterations and a large array of different chronic conditions, including cardio-cerebrovascular diseases, heart failure, chronic kidney disease, osteoarticular diseases, depression, sleep apnea, and neoplasms of the gastrointestinal tract. Within each BMI class, the presence of IFG, and to a greater extent DM, identified subgroups of individuals with a marked increase in the risk of concomitant chronic conditions.

**Conclusion:**

Addressing the double burden of excess weight and hyperglycemia represents an important challenge and a healthcare priority.

## Introduction

The continuing growth in the prevalence of overweight and obesity is a cause of serious concern in all regions of the world, and the phenomenon is increasingly taking the form of a global pandemic. In 2015, a total of 603.7 million adults were obese [1]. Since 1980, the prevalence of obesity has doubled in more than 70 countries and has risen continuously in most other countries. In 2015, the high body mass index (BMI) caused 4.0 million deaths globally, nearly 40% of which occurred in non-obese people (BMI < 30 kg/m^2^). More than two thirds of high BMI-related deaths were due to cardiovascular disease [1].

In Europe, the prevalence of obesity has tripled in many countries since the 1980s and continues to grow at an alarming rate. According to recent WHO estimates, one in two citizens in Europe is overweight or obese, while one in five suffers from obesity [2]. Among 11-year-old children, one in three is overweight or obese [2]. The prevalence of excess weight among men over the age of 20 exceeds 60% in several European countries, including Greece, the United Kingdom, Ireland, Germany, Portugal, Spain and Finland, while Italy stands at a percentage just under 60% [3]. Specifically concerning Italy, data from the National Institute of Statistics (ISTAT) reveals a growth of approximately two million overweight individuals and over one million obese individuals between 2001 and 2010. In 2016, over 23 million adults (45.9%) were classified as overweight or obese, with 17.9 million overweight and 5.2 million obese [4]. Excess weight carries significant clinical, social, and economic implications due to its connection with numerous health conditions such as diabetes, cardiovascular diseases, respiratory diseases, certain types of cancer, and osteoarticular diseases [5].

Obesity and type 2 diabetes are metabolic disorders whose prevalence has increased significantly in recent decades. In this scenario, the classic risk factors are joined by others linked to the evolution of economic pressure and consumer demand. The term diabesity defines the pathophysiological connection between overweight and type 2 diabetes [6]. The strong association between excess weight and diabetes, and their interplay in increasing the cardiovascular risk is well known, and the majority of individuals with type 2 diabetes are either overweight or obese [7]. Together, they increase the individuals’ mortality risk sevenfold [8].

However, end-organ complications of hyperglycemia may be present even before the development of overt diabetes. Indeed, pre-diabetes is associated with an increased incidence of diabetes-specific microvascular and macrovascular complications [9] and an increase in cardiovascular events and all-cause mortality [10], compared with age-matched and BMI-matched people with normal glucose tolerance (NGT). In 2021, the worldwide number of individuals with impaired fasting glucose (IFG) is estimated to be 319 million adults, or 6.2% of the global adult population [11]. Recent projections foresee that in 2045 this number is expected to further increase to an estimated of 441 million adults or 6.9% of the global adult population. In Italy, according to International Diabetes Federation estimates, in 2021 there were 4.47 million people aged 20–79 years with known diabetes, 1.5 million people with unknown diabetes, and 162 million people with IFG [11].

Measuring and addressing the interplay between excess body weight and hyperglycemia is then paramount from a healthcare policy perspective. The purpose of this study was then to evaluate the prevalence of chronic comorbidities according to BMI classes and, for the first time, assess the interplay between various levels of excess body weight and blood glucose abnormalities associated with increasing prevalence of major chronic diseases, using population data deriving from a large sample of medical records collected by Italian General Practitioners (GPs).

## Methods

### Data used

The study is based on data from the Health Search/IQVIA Health LPD Longitudinal Patient Database (HS), an Italian general practice registry, which collects data obtained from electronic clinical records (ECRs) of patients mostly aged 14+ assisted by a group of 800 GPs, homogeneously distributed across all Italian regions (Appendix D). GPs voluntarily agree to collect patients’ information and to attend training courses for data entry [12]. Furthermore, to be considered for participation in epidemiological studies, all recruited GPs need to meet “up to standard” quality criteria pertaining to the levels of coding, prevalence of well-known diseases, mortality rates, and years of recording activity [13]. The database complies with European Union guidelines on the use of medical data for research and has been previously demonstrated to be a valid data source for scientific research [14]. To guarantee the quality of the data and the reliability of the results, from the full list of patients we have selected only active patients, defined as individuals who were still alive and had not revoked their GP.

A key feature of the HS database is that it includes all patients registered in the GP rosters, which avoids the possibility of selection bias based on health status. The database contains patient demographic data (age, sex, region of residence) that are linked through an encrypted patient code with their medical records (diagnoses, prescribed tests, and tests results), drug prescription information (medication name, date of filled prescription, and number of days’ supply), self-reported hospital admissions, and date of death. Although GPs collect the information on daily basis, for the purpose of this analysis the information has been aggregated at year level. Whenever information on specific variables was recorded more than once in a year (i.e., diagnostic test values and BMI levels), we have averaged these values over the year, thus resulting in a single observation per year.

The data included in the Health Search/IQVIA Health LPD Longitudinal Patient Database are representative at national level. Of note, the Italian National Institute of Statistics (ISTAT) uses these data to complement the information collected with the annual national health survey [15]; moreover, the Italian Drug Agency has routinely used the Health Search database as a source for the National Report on the Use of Drugs in Italy since 2004 [16, 17]. Furthermore, researchers have observed “a high degree of overlap between the population represented in the Health Search database with respect to what is reported by ISTAT” [18].

### Sample size

The original sample contained a total of 1,517,682 individuals over the period 2004–2018. For the purposes of our study, we selected people in the age range 18–95 years, thus leading to a sample of 1,498,598 individuals. Finally, we have discarded outliers and mis-recordings of diagnostic test variables (i.e. negative and implausible values). For the year 2018 this selection process produced a final sample of 991,917 individuals.

### Data imputation strategy

Since the dataset presented missing data in BMI, a statistical imputation strategy was applied. The lack of BMI records across patients and over time is rather common: if there are no medical and clinical reasons to measure height and weight, GPs tend not to routinely measure them at each visit. A second problem that interferes with the regular collection of BMI data is related to a selection process for which, whenever patients are not medically and clinically problematic in terms of BMI, GPs tend to collect this information less frequently. Therefore, one of the main threats to our analysis is the presence of missing values on BMI measurements, especially for the healthy population. Nonetheless, the sample still presents information on BMI levels (over time) for this subgroup. For this reason, although the analyses were conducted only on data referring to 2018, we exploited the longitudinal characteristic of our sample to estimate a more robust model that could be later used to impute the missing information in 2018. Hence, assuming that the sample selection generated by the missingness is driven by observable characteristics such as health status and age, we made an attempt to lessen the negative impact of the ascertainment bias on our analysis. Our objective was not to impute the BMI level to each subject, a too challenging task, but at least try to infer the BMI class to which each subject belongs. To this aim, we exploited a pooled ordered Probit panel data model with sample selection in which the response is an ordered categorical variable indicating the BMI classes [19]. The latter have been constructed according to the following classification: (i) normal weight [18.5–24.99], (ii) overweight [25.0–29*.*99], (iii) obese class 1 [30.0–34.99]; (iv) obese class 2 [35.0–39.99], (v) obese class 3 [≥ 40]. For the sake of brevity but without any loss of information, we do not report the underweight class (BMI below 18.49) as not relevant for our analysis. The details of the imputation strategy used are reported in Appendix A.

### Variable construction

The study population was classified according to BMI levels (expressed as kg/m^2^) in the following groups: normal weight (BMI between 18.5 and 24.99), overweight (BMI between 25 and 29.99), obesity class 1 (BMI between 30 and 34.99), obesity class 2 (BMI between 35 and 39.99), obesity class 3 (BMI ≥ 40) [20]. The population was also classified according to glucose metabolism status into three classes: normal glucose tolerance (NGT; no diagnosis of diabetes mellitus over the period 2004–2018, fasting blood glucose < 100 mg/dl), impaired fasting glucose (IFG; no diagnosis of diabetes mellitus over the period 2004–2018, fasting blood glucose between 100 and 125 mg/dl), diabetes mellitus (DM; diagnosis of diabetes mellitus over the period 2004–2018).

All diagnoses in our database are coded according to the International Classification of Disease, ninth revision (ICD-9 CM) [21]. The list of codes used to identify comorbidities is reported in Appendix B. Among neoplasms, those affecting the digestive apparatus were selected (esophagus, stomach, intestine, colon, rectum, liver, gallbladder, pancreas).

This study did not require ethical approval and consent to participate.

## Results

### The burden of overweight and obesity

All data summarized in Table 1 are represented as mean and standard deviation (continuous variables) or percentages (categorical variables). Overall, data relative to 991,917 adults were analyzed. The prevalence of overweight was 39.4%, while the prevalence of obesity was 11.1% (class 1: 7.9%, class 2: 2.3%, class 3: 0.9%). Participants’ characteristics, overall and by BMI classes, are reported in Table 1, while Table 4 in Appendix C reports t-tests on mean differences across the several variables, proving that all difference discussed in this section are statistically significant at 1% level. Compared to the overall sample, people with normal weight had a younger age and a higher prevalence of females. Among individuals with excess weight, those with class 3 obesity had the lowest mean age, while those with obesity class 1 tended to be older. The prevalence of females increases from 38.8% among overweight individuals up to 66.8% among severely obese individuals.

**Table 1:** Characteristics of the study population, overall and by BMI classes

Characteristics	Overall	Normal weight	Overweight	Obesity class 1	Obesity class 2	Obesity class 3
Number of observations	991,917	476,571	390,979	78,413	22,887	8752
Age (years)	52.5 ± 18.7	44.5 ± 17.3	60.30 ± 16.5	62.0 ± 15.8	60.6 ± 15.7	58.2 ± 15.4
Gender (% females)	51.9	61.8	38.8	48.1	59.0	66.8
Fasting blood glucose (mg/dl)	102.6 ± 28.3	94.3 ± 20.8	104.8 ± 28.1	113.0 ± 34.3	116.9 ± 38.3	118.7 ± 40.3
HbA1c (mmol/mol)	45.2 ± 12.8	42.5 ± 12.1	45.2 ± 12.5	46.8 ± 13.9	47.9 ± 13.9	47.4 ± 14.4
Systolic blood pressure (mmHg)	131.3 ± 14.3	127.8 ± 14.6	132.6 ± 13.8	134.0 ± 13.5	134.7 ± 13.6	134.9 ± 14.1
Diastolic blood pressure (mmHg)	78.4 ± 8.3	77.1 ± 8.3	78.8 ± 8.1	79.5 ± 8.1	80.1 ± 8.2	80.8 ± 8.5
Total cholesterol (mmol/l)	5.13 ± 1.01	5.23 ± 0.99	5.13 ± 1.01	4.99 ± 1.03	4.92 ± 1.02	4.86 ± 0.95
HDL cholesterol (mmol/l)	1.45 ± 0.38	1.58 ± 0.40	1.40 ± 0.35	1.32 ± 0.33	1.30 ± 0.32	1.29 ± 0.32
LDL cholesterol (mmol/l)	3.08 ± 0.88	3.14 ± 0.87	3.08 ± 0.89	2.97 ± 0.89	2.90 ± 0.88	2.87 ± 0.90
Triglycerides (mmol/l)	1.38 ± 0.85	1.20 ± 0.76	1.44 ± 0.87	1.63 ± 0.96	1.63 ± 0.96	1.60 ± 0.87
Glycemic status (%):						
NGT IFG DM	86.9 4.2 8.9	95.2 2.4 2.4	82.4 5.6 12.0	67.6 7.9 24.5	61.7 6.8 31.5	57.7 5.4 36.8
Hypertension (%)	32.9	15.5	45.4	65.2	70.0	72.5
Dyslipidemia (%)	20.3	11.9	27.2	34.7	31.5	26.4
Coronary heart disease (%)	1.95	0.74	2.95	4.09	3.72	2.45
Cerebrovascular disease (%)	5.16	2.63	7.15	9.82	8.78	7.16
Heart failure (%)	4.10	1.43	5.50	10.32	12.44	14.05
Osteoarticular diseases* (%)	6.87	2.81	9.41	15.16	18.05	19.10
Depression (%)	6.18	4.78	7.05	8.67	9.57	11.09
Chronic kidney disease (%)	2.37	1.11	3.00	5.65	6.00	6.11
Cancer** (%)	1.12	0.81	1.36	1.72	1.44	1.03
Sleep apnea (%)	1.67	0.59	1.88	4.52	7.62	11.95
PCOS*** (%)	1.49	1.87	0.77	1.15	1.30	1.59
No. of comorbidities (%):						
0 1–3 > 3	59.2 39.2 1.6	77.4 22.0 0.6	45.8 52.2 2.0	27.1 68.5 4.4	23.1 71.5 5.5	20.8 73.0 6.2

*NGT* normoglycemia, *IFG* impaired fasting glucose, *DM* diabetes mellitus

*Hip and knee osteoarthrosis

**Esophagus, stomach, intestine, colon, rectum, liver, gallbladder, pancreas

***Polycystic ovary syndrome

Average values of fasting blood glucose, HbA1c, blood pressure, and triglycerides linearly increased with increasing BMI, while an opposite trend was observed for HDL cholesterol, LDL cholesterol, and total cholesterol.

**Fig. 1:**
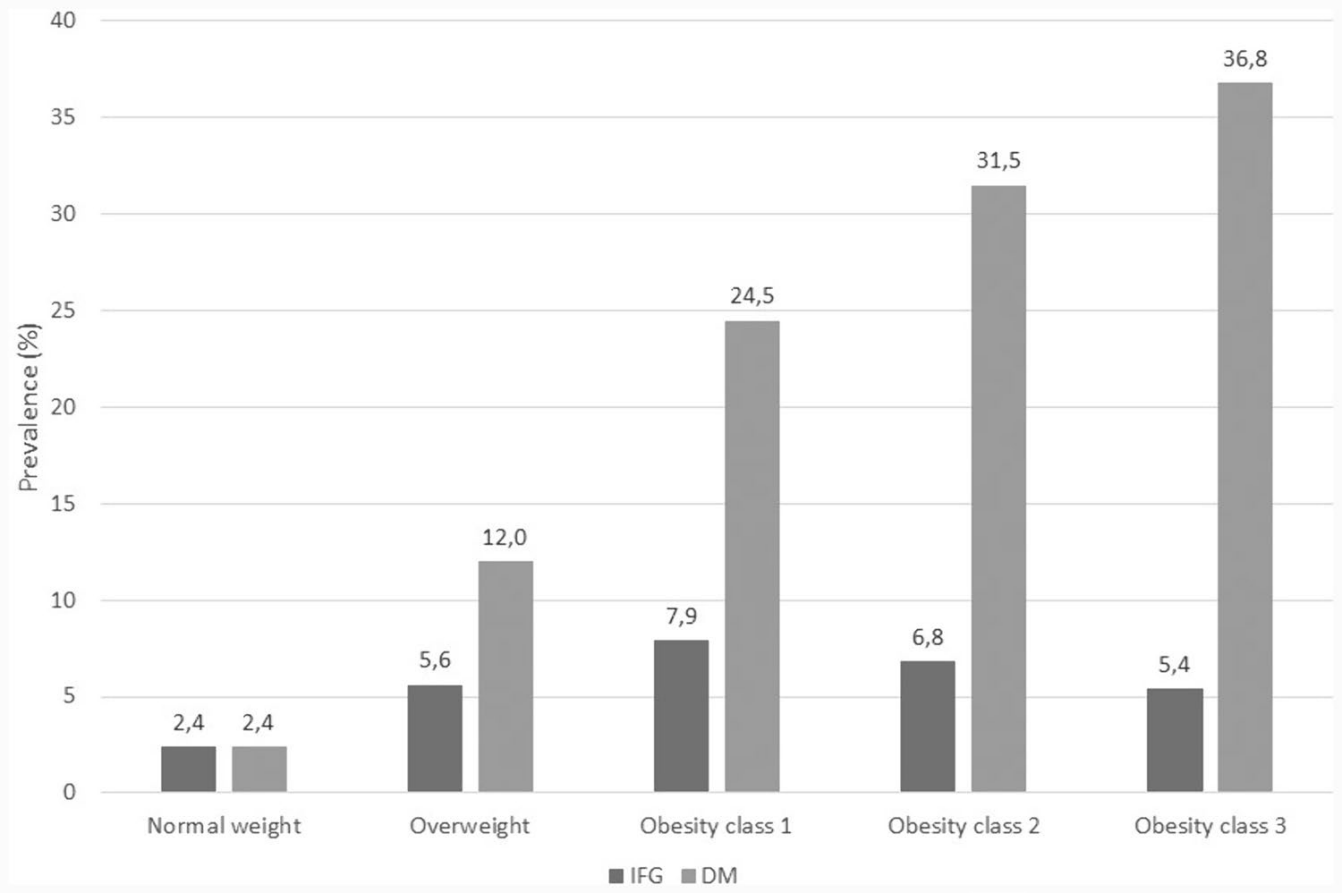
Prevalence of glucose metabolism alterations by BMI classes

The prevalence of glucose metabolism alterations markedly increased with increasing levels of BMI (Fig. 1). In the whole population, the prevalence of diabetes was 8.9%; compared to individuals with normal weight, the prevalence of diabetes was five times higher among those with overweight, ten times higher among those with obesity class 1, 13 times higher among those with obesity class 2 and 15 times higher among those with obesity class 3. Overall, the prevalence of IFG was 4.2%, being the lowest in individuals with normal weight (2.4%) and the highest among those with obesity class 1 (7.9%).

**Fig. 2:**
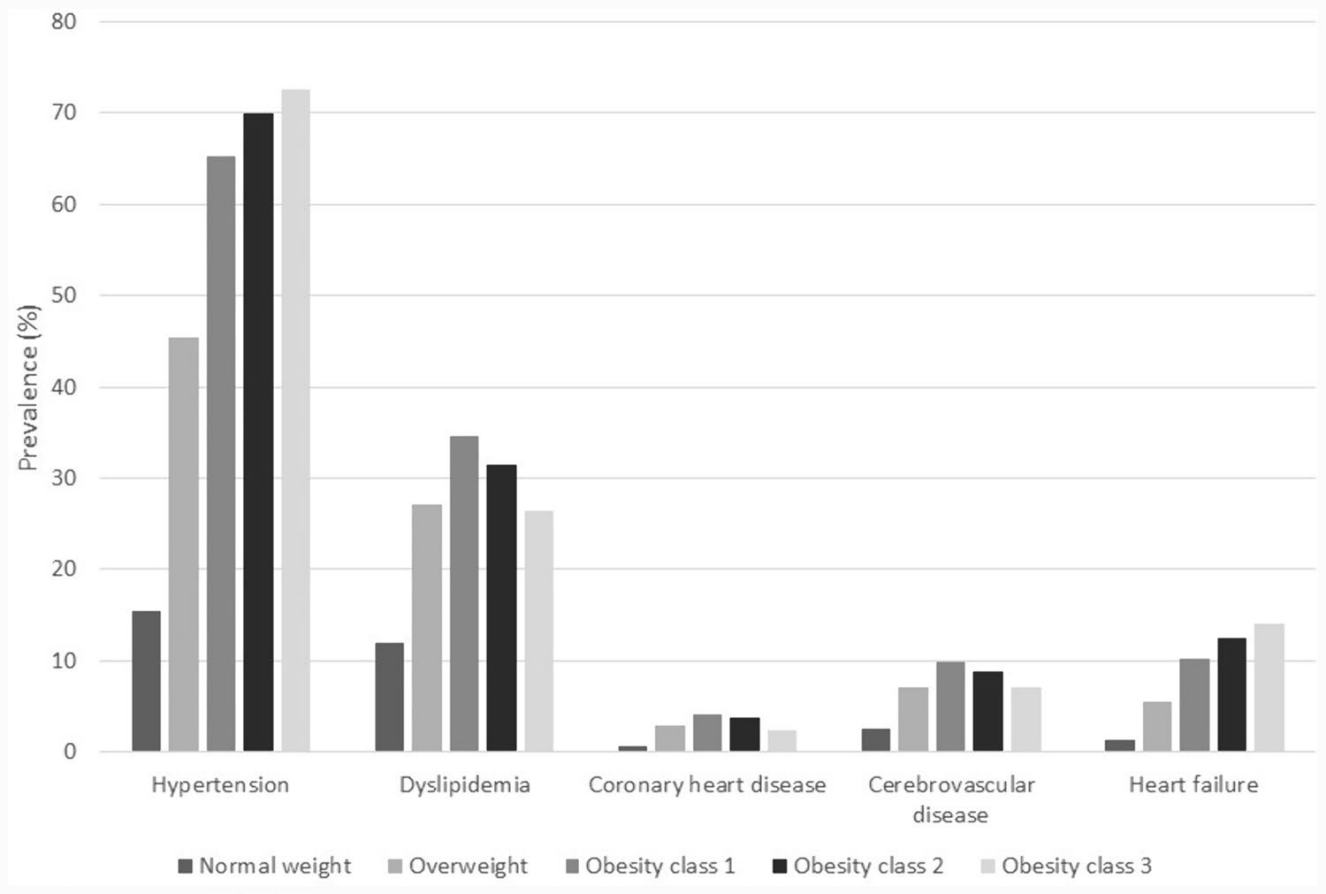
Prevalence of cardiovascular comorbidities by BMI classes

The prevalence of major cardiovascular risk factors and cardiovascular events is also associated with BMI (Fig. 2). In particular, the proportion of people affected by hypertension increased linearly with BMI, to reach 72.5% among severely obese individuals. The prevalence of dyslipidemia shows a pattern similar to that of IFG, being the lowest among people with normal weight, and the highest among those with obesity class 1. Compared to people with normal weight, the prevalence of coronary heart disease was 4 times higher among those overweight and 5.5 times higher among those in obesity class 1, while it slightly decreased among very severely obese individuals. Similarly, the prevalence of cerebrovascular disease was about 3 times higher in individuals with any level of excess weight, being the highest among those with obesity class 1. The prevalence of heart failure dramatically increased with increasing BMI levels; in particular, compared to normal weight, obesity class 3 was associated with a ten times higher prevalence of heart failure.

**Fig. 3:**
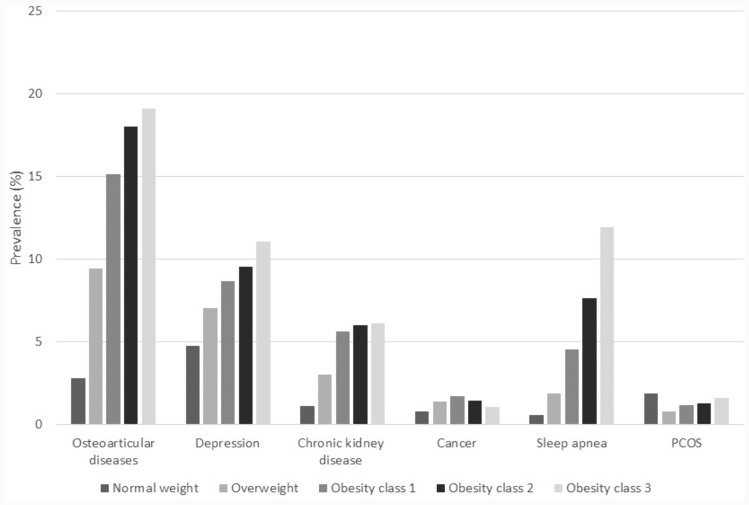
Prevalence of selected chronic diseases by BMI classes

Among the other chronic conditions considered, osteoarticular diseases, depression, chronic kidney disease and sleep apnea markedly increased with BMI (Fig. 3). Compared to individuals with normal weight, those with obesity class 3 had a seven times higher prevalence of osteoarticular disease, a 5.5 times higher prevalence of chronic kidney disease and a 20-fold increase in the prevalence of sleep apnea. The prevalence of neoplasms was highest among people with obesity class 1. Among individuals with normal weight, 77.4% had none of the investigated comorbidities; on the other hand, one in two overweight individuals and around 70% of obese individuals had one or more comorbidities.

### Interplay between excess weight and glucose metabolism alterations

**Fig. 4:**
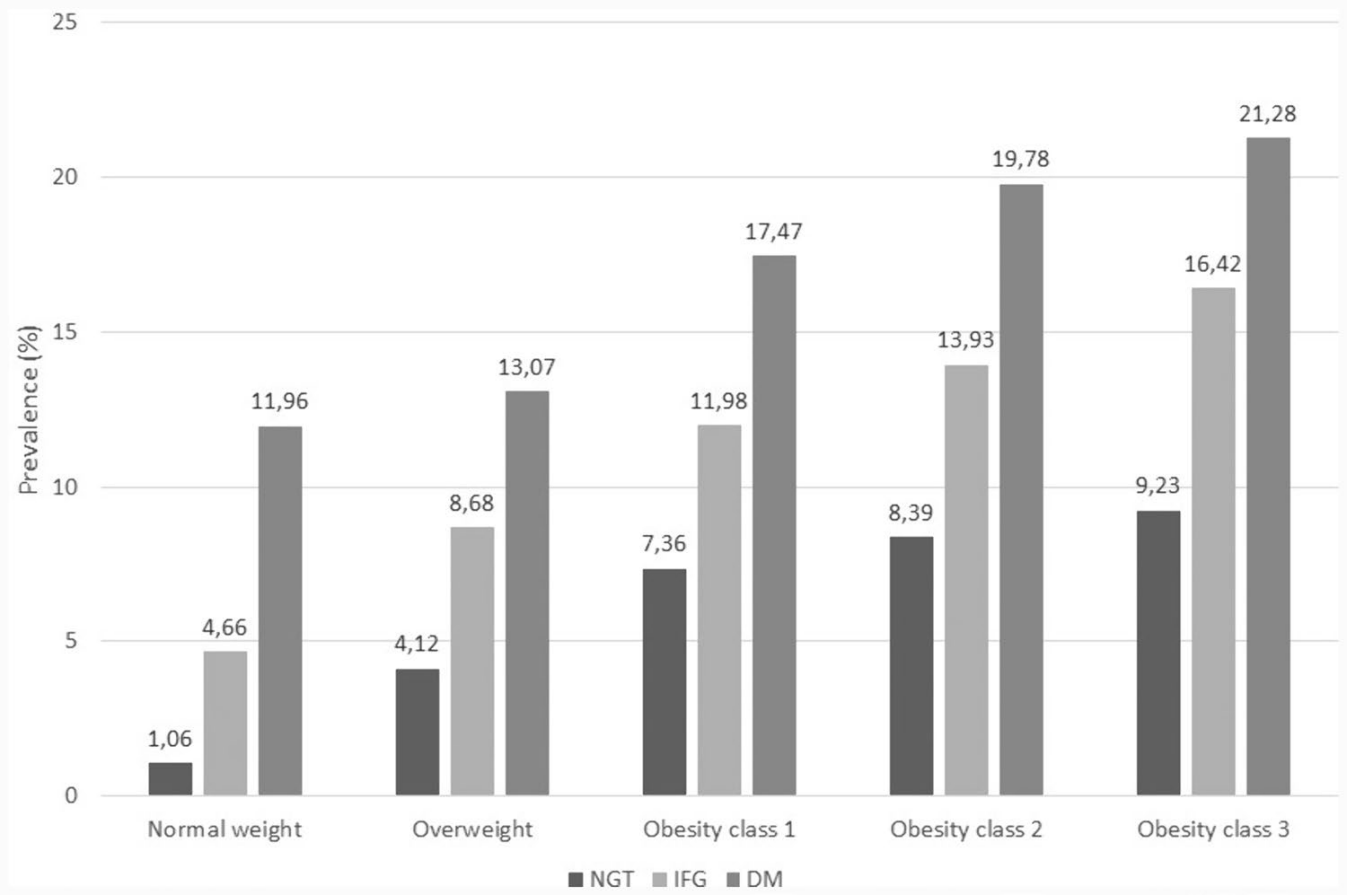
Prevalence of heart failure by BMI and glucose metabolism classes

The study population was also analyzed according to glycemic status within each BMI category. All results are reported in Table 2. Furthermore, in Table 4 in Appendix C we report t-tests on mean differences, proving that all difference discussed in this section are statistically significant at 1% level. The presence of IFG identified, within each class of BMI, a subgroup of individuals with a marked increase in the risk of all the comorbidities investigated, with the only exception of depression, CKD and cancer among very severely obese individuals and PCOS in all BMI classes. In particular, for all BMI classes, IFG was associated with a noticeable increase in the prevalence of hypertension and dyslipidemia, as well as in the proportion of individuals affected by coronary heart disease, cerebrovascular disease, and heart failure. The presence of diabetes further increased the risk of all chronic conditions investigated. Of note, the prevalence of coronary heart disease and cerebrovascular disease among individuals with diabetes was very similar across BMI classes, with the exception of a lower prevalence among very severely obese people, suggesting that diabetes per se, rather than excess weight, plays the major role in increasing cardiovascular risk. Similarly, the prevalence of chronic kidney disease was markedly higher in the presence of diabetes, but without major differences across BMI classes. On the other hand, the percentage of people with heart failure was strikingly increased in the presence of DM, and linearly increased with increasing BMI, suggesting ad additive effect of excess body weight and blood glucose alterations (Fig. 4). Osteoarticular diseases and depression were also more frequently registered in the presence of diabetes, and their prevalence increased with increasing BMI levels.

**Table 2:** Prevalence of comorbidities according to BMI classes and glycemic status (%)

Comorbidities	Normal weight			Overweight			Obesity class 1			Obesity class 2			Obesity class 3		
	NGT	IFG	DM	NGT	IFG	DM	NGT	IFG	DM	NGT	IFG	DM	NGT	IFG	DM
Hypertension	13.30	45.88	65.61	39.84	64.39	72.38	56.93	75.75	83.22	60.63	80.27	84.93	62.35	82.32	86.41
Dyslipidemia	10.48	36.89	39.53	24.11	42.99	39.57	29.25	45.67	44.86	25.27	40.10	40.94	19.85	28.84	35.92
Coronary heart disease	0.50	2.96	7.19	2.06	5.43	7.63	2.52	5.68	7.67	2.10	3.80	6.83	1.48	1.68	4.00
Cerebrovascular disease	2.01	9.40	18.32	5.45	12.38	15.80	6.77	12.61	16.74	5.89	10.06	13.90	5.14	7.16	10.20
Heart failure	1.06	4.66	11.96	4.12	8.68	13.07	7.36	11.98	17.47	8.39	13.93	19.78	9.23	16.42	21.28
Osteoarticular diseases*	2.41	7.62	12.30	8.30	13.78	14.62	13.21	17.52	19.45	15.28	21.41	22.61	15.16	21.89	24.75
Depression	4.51	8.55	10.81	6.64	7.90	9.38	7.79	9.06	10.85	8.48	9.99	11.56	9.88	9.05	13.31
Chronic kidney disease	0.82	3.46	9.48	2.11	4.49	8.16	3.69	6.05	10.71	3.43	5.74	11.12	3.76	3.79	10.11
Cancer**	0.67	2.58	4.05	1.05	2.06	3.00	1.34	1.91	2.63	0.88	1.61	2.40	0.67	0.63	1.64
Sleep apnea	0.55	1.48	1.19	1.66	3.20	2.65	3.91	6.23	5.58	6.26	10.32	9.63	10.00	12.42	14.80
PCOS***	1.20	0.35	0.37	0.32	0.10	0.25	0.73	0.19	0.23	0.95	0.52	0.42	1.24	0.84	0.78
No. of comorbidities:															
0 1–3 > 3	79.9 19.8 0.4	43.1 55.2 1.8	23.3 70.6 6.1	51.4 47.3 1.3	25.6 71.1 3.3	19.0 75.0 6.0	34.8 62.5 2.7	16.4 78.5 5.1	10.5 80.7 8.8	31.7 65.1 3.2	13.5 80.5 6.0	9.5 80.6 9.9	29.9 66.3 3.8	13.5 80.2 6.3	8.1 89.0 9.9

*NGT* Normoglycemia, *IFG* impaired fasting glucose, *DM* diabetes mellitus

*Hip and knee osteoarthrosis

**Esophagus, stomach, intestine, colon, rectum, liver, gallbladder, pancreas

***Polycystic ovary syndrome

## Discussion

### Major findings

The analysis of a large clinical database of primary care in Italy confirmed that excess body weight is extremely common, affecting half of the adult population. Our findings are il line with recent WHO estimates, according to which one in two citizens in Europe is overweight or obese [2]. We also confirmed the elevated clinical burden associated with excess weight: both overweight and obesity were associated with an increasing prevalence of a large array of different chronic conditions, including cardiovascular diseases, chronic kidney disease, osteoarticular diseases, depression, sleep apnoea, and neoplasms of the gastrointestinal tract.

Glucose metabolism alterations are particularly linked to overweight and obesity: diabetes was registered in one in seven people with normal weight, one in four overweight individuals, one in three individuals with obesity class 1 and 2, and four in ten severely obese individuals. The presence of diabetes strongly influenced the likelihood of suffering from other chronic conditions. In addition, a pre-diabetes status, as indicated by the presence of impaired fasting glucose, was also associated with an elevated risk of major comorbidities, suggesting a continuum in the risk related to glucose abnormalities. Overall, our data show that the prevalence of the typical comorbidities associated with overweight and obesity is not uniform within each BMI class, and that glucose metabolism alterations help in identifying subgroups with a substantial increase in the risk of other chronic conditions. Of note, people with very severe obesity showed lower levels of total and LDL cholesterol and lower rates of some concomitant diseases, particularly coronary heart disease and cerebrovascular disease, as compared to individuals with obesity class 1 and 2. While these findings can suggest a greater attention to control major cardiovascular risk factors in the presence of more elevated BMI, the existence of a survivor bias cannot be excluded. In fact, mortality rates among very severely obese individuals tend to be higher [22], thus determining a selection of the less severe cases.

In our study, the prevalence of chronic heart failure (CHF) clearly increased with increasing BMI. Furthermore, within each BMI class, a strong trend of increasing prevalence was observed in relation to the presence of IFG and diabetes. CHF is an important condition to monitor as it affects over 64 million people in the world, and prevalent cases and years lived with disability have increased by 91.9% and 106.0% from 1990, respectively [23]. Previous studies have demonstrated that even metabolically healthy obese individuals (i.e. without hypertension, dyslipidemia, and dysglycemia) are at higher risk of heart failure [24]; furthermore, heart failure prevalence in normal weight, overweight, and obese individuals increased with increasing number of metabolic abnormalities [24]. In a pooled population-based cohort study involving 24,675 participants without a history of heart failure, 21% of incident cases of heart failure were attributable to obesity among individuals under 55 years of age and 13% among the elderly ones [25]. The association between obesity and heart failure was confirmed in a metanalysis of 29 studies, showing a ‘J curve’ relationship between BMI and risk of heart failure, with maximum risk in the morbidly obese (OR = 1.73 (95% CI 1.30–2.31) [26]. The metanalysis also documented an improvement in cardiac indices after intentional weight loss.

Heart failure and chronic kidney disease deserve a particular consideration. In our study, the prevalence of chronic kidney disease and heart failure clearly increased with increasing BMI. These conditions are closely interconnected, leading to the connotation of “cardiorenal syndrome” [27], and are related to hyperglycemia. The global burden of chronic kidney disease is considerable and growing: it is estimated that around 10% of adults worldwide are affected, resulting in 1.2 million deaths and 28 million years of life lost each year [28]. The combined effect of body mass index and metabolic status on the risk of prevalent and incident chronic kidney disease was documented in a metanalysis involving over 180,000 participants [29]. The study showed that, compared with metabolically healthy normal weight individuals, metabolically healthy obese individuals showed a 23.5% increased risk for CKD (RR = 1.235; 95%CI: 1.027–1.484). Metabolically abnormal groups had much higher risk for CKD, with RR of 1.572 (95%CI: 1.373–1.801), 1.652(95%CI: 1.139–2.397) and 1.898(95%CI: 1.505–2.395) for unhealthy normal weight, overweight and obese individuals, respectively. A more recent metanalysis of nine prospective cohort studies with 128,773 participants confirmed that metabolically healthy overweight individuals were at increased risk for CKD (RR = 1.34; 95% CI: 1.20–1.51). In metabolically healthy obese participants, the risk of CKD further increased (RR = 1.55; 95% CI: 1.34 to 1.79), while metabolically unhealthy obese individuals showed the highest risk (RR = 2.13; 95% CI: 1.66–2.72) [30].

Also, the prevalence of those conditions typically associated with excess body weight, such as osteoarticular diseases and sleep apnea, linearly increase with increasing body weight.

### Implications for clinical practice

Our study provides an up-to-date estimate of the clinical burden of excess weight in Italy, and strongly suggests the need to intervene to limit the growth of the obesity pandemic. The study clearly shows that even moderate increases in body weight, in the range of overweight, are associated with an increased prevalence of many chronic conditions, thus suggesting the need to intervene in a timely and effective manner to counteract weight gain. The study also shows that the concomitance of excess weight and glucose metabolism alterations, even before the stage of overt diabetes, further increases the risk of comorbidities. A proactive approach is thus needed to identify glucose metabolism alterations and to address them with specific lifestyle and pharmacological interventions. These findings are particularly important for primary care, which represents the forefront of the fight against obesity, diabetes, and related comorbidities.

### Strengths and limitations

Our study has strengths and limitations. Among the strengths, it should be mentioned the very large study sample, representative of the Italian adult population, providing a realistic picture of the burden associated with different levels of excess weight. The good data quality represents another important aspect, allowing a reliable estimate of the prevalence of chronic conditions associated with overweight and obesity.

The study also has limitations related to the intrinsic nature of the data, reflecting the real-world practice of GPs. In particular, missing data could, at least in theory, affect the precision of the estimates. We have tried to minimize this problem by applying validated missing imputation techniques. In this respect, the consistency of our findings with data from other epidemiological sources provides reassurance regarding the reliability of our findings. Furthermore, it is acknowledged that waist circumference represents a more accurate measure of visceral adiposity and obesity-related health risk compared to BMI [31]. However, waist circumference was seldom reported in clinical records, precluding the possibility to use this measure in our study.

## Conclusions

In conclusion, one in two Italian adults have excess body weight, and one in ten is obese. This has important health consequences, as documented by the increase in prevalence of a large array of chronic conditions associated with increasing levels of BMI. Blood glucose alterations are particularly common among overweight and obese individuals, and are associated with a higher likelihood of suffering from other comorbidities. Addressing the double burden of excess weight and hyperglycemia represents an important challenge and a healthcare priority.

## Data Availability

The data that support the findings of this study are available from Health Search but restrictions apply to the availability of these data, which were used under license for the current study, and so are not publicly available. Data are however available from the authors upon reasonable request and with permission of Health Search.

## Appendix A. Missing imputation techniques adopted

Within the HS database, over the 2004–2018 period, there were 641,849 individuals with at least one value of BMI recorded. Our statistical model to recover information on BMI via statistical imputation can be represented as follow:

1 $${y}_{j,it}^{*} ={{\varvec{x}}}_{j,it}{\beta }_{j}+{\epsilon }_{j,it} j=\mathrm{1,2}$$

2 $${y}_{1,it}= I({y}_{1,it}^{*}>0)$$

3 $${y}_{2,it}=\sum_{h=1}^{H}hI({\alpha }_{h}<{y}_{2,it}^{*}\le {\alpha }_{h+1}) if {y}_{1,it}=1$$

where ${y}_{1,it}^{*}$ and ${y}_{2,it}^{*}$ represent, for each subject *i* and year *t*, continuous latent variables for the selection process and the BMI outcome, respectively, the ${{\varvec{x}}}_{1,it}$ and ${{\varvec{x}}}_{2,it}$ are vectors of exogenous of individual characteristics, such as age, gender and region of residence, the fraction of individuals whose BMI was effectively recorded for year* t* at GP level and year fixed-effects. ${{\varvec{x}}}_{2,it}$ also includes a vector of selected diseases indicators related to obesity (cerebrovascular diseases, coronary diseases, heart failure, peptic ulcer disease, diabetes mellitus) and solid tumor (esophagus, stomach, intestine, colon, rectum, liver, gallbladder, pancreas). Finally, the ${\epsilon }_{1,it}$ ${\epsilon }_{2,it}$ are idiosyncratic errors, ${\beta }_{j}, j=\mathrm{1,2}$, are the vectors of parameters to be estimated.

As in a standard ordered probit model, the (latent) BMI ${y}_{2,it}^{*}$ is assumed to be linked to the observed categorical variable ${y}_{2,it}$ (BMI classes) through the observation rule (3), where $\delta =({\delta }_{0},\dots ,{\delta }_{H})$, with ${\delta }_{h}\le {\delta }_{h+1}$ is a vector of unknown thresholds that partition ${y}_{2,it}^{*}$ into $H+1$ mutually exclusive BMI classes. Due to the missing data (selection mechanism), the ${y}_{2,it}$ variable is observed only for the subsample of observation for which ${y}_{1,it}=1$ i.e. the selected sample.

Identification of the unknown parameter vectors ${\beta }_{j}, j=\mathrm{1,2}$ and $\delta$ requires an exclusion restriction in the selection Eq. (2), that ${{\varvec{x}}}_{1,it}$ must contain at least one variable that is not contained in ${{\varvec{x}}}_{2,it}$.^1^

We argue that the selection mechanism is driven by health status, thus we consider as exclusion restriction two binary indicators for being healthy and under 40 years.

The joint distribution of ${\epsilon }_{1,it}$ ${\epsilon }_{2,it}$ is assumed to be bivariate normal with zero mean, ${{\text{Var}}(\epsilon }_{1i})= {\text{Var}}({\epsilon }_{2i})=1$, and $Cov\left({\epsilon }_{1,it} {\epsilon }_{2,it}\right)=\rho .$ We estimated model (1)-(3) by pooling the unbalanced panel of 1,498,598 individuals aged 18–95 years over the 15 year period.^2^ We then used the estimated parameters to estimate the probability that ${y}_{2,it}=h$ given that ${y}_{2,it}$ is not selected, that is:

4 $${\text{Pr}}({y}_{2,it}\widehat{={h|y}_{1,it}}= 0)=\frac{{\text{Pr}}{(y}_{2,it}\widehat{={h,y}_{1,it}}= 0)}{{\text{Pr}}(\widehat{{y}_{1,it}}= 0)}$$

where

5 $${\text{Pr}}\left({y}_{2,it}\widehat{={h,y}_{1,it}}= 0\right)={\Phi }_{2}\left(-{{\varvec{x}}}_{2,it}\widehat{{\beta }_{2}, }{\delta }_{h}-{{\varvec{x}}}_{1,it}\widehat{{\beta }_{1}, }\rho \right)-{\Phi }_{2}\left(-{{\varvec{x}}}_{2,it}\widehat{{\beta }_{2}, }{\delta }_{h-1}-{{\varvec{x}}}_{1,it}\widehat{{\beta }_{1}, }\rho \right)$$

with ${\Phi }_{2}$ the cumulative bivariate normal distribution function (with mean [0,0]*'*) and the probability that the outcome ${y}_{2,it}=h$ is not selected is${\text{Pr}}\left(\widehat{{y}_{1,it}}=0\right)=1-\Phi \widehat{({{\varvec{x}}}_{1,it}\widehat{{\beta }_{1}, }})$. We then assigned the missing BMI class to subject *i* in year *t* accordingly, i.e. ${\widehat{y}}_{2,it}=h$ if ${\text{Pr}}{(y}_{2,it}\widehat{={h|y}_{1,it}}= 0)<{\text{Pr}}\left({y}_{2,it}\widehat{=j|}{y}_{1,it}= 0\right)\forall j\ne h$.

## Appendix B. ICD-9 CM codes used to identify comorbidities

**Table:**

Comorbidity	ICD-9 CM codes
Diabetes mellitus	250
Coronary heart disease (myocardial infarction, coronary reperfusion/revascularization)	410, 412, v45.81, v45.82
Cerebrovascular disease (stroke, transient ischemic attack, carotid artery revascularization)	431, 433.00, 433.01, 433.11, 433.21, 433.31, 433.81, 433.91, 434.01, 434.11, 434.91, 435, 436, 438, v45.89
Heart failure	428, 402.0, 402.01, 402.11, 402.91, 404.00, 404.03, 404.11, 404.13, 404.91, 404.93
Osteoarticular diseases (hip and knee osteoarthrosis)	715.35, 715.95, 715.36, 715.96
Depression	311, 296.2, 296.3
Chronic kidney disease	585
Cancer (esophagus, stomach, intestine, colon, rectum, liver, gallbladder, pancreas)	150, 151, 152, 153, 154, 155, 156, 157
Sleep apnea	780.51, 780.53, 780.57
PCOS	256.4

## Appendix C

See Appendix Tables

**Table 3:** T-test on mean differences for values reported in Table 1

	Normal vs Overweight	Normal vs Obese I	Normal vs Obese II	Normal vs Obese III
Age	− 15.85***	− 17.58***	− 16.19***	− 13.78***
Prevalence female (%)	22.97***	13.72***	2.775***	− 5.006***
Fasting blood glucose (mg/dl)	− 10.54***	− 18.74***	− 22.61***	− 24.41***
HbA1c (mmol/mol)	− 2.716***	− 4.358***	− 5.452***	− 4.908***
Systolic blood pressure (mmHg)	− 4.794***	− 6.231***	− 6.852***	− 7.070***
Diastolic blood pressure (mmHg)	− 1.611***	− 2.367***	− 2.995***	− 3.603***
Total cholesterol (mmol/l)	0.0995***	0.239***	0.301***	0.366***
HDL cholesterol (mmol/l)	0.173***	0.254***	0.278***	0.284***
LDL cholesterol (mmol/l)	0.0554***	0.171***	0.235***	0.271***
Triglycerides (mmol/l)	− 0.241***	− 0.380***	− 0.435***	− 0.402***
Hypertension (%)	− 29.87***	− 49.68***	− 54.44***	− 57.01***
Dyslipidemia (%)	− 15.28***	− 22.71***	− 19.55***	− 14.47***
Coronary heart diseases (%)	− 2.210***	− 3.356***	− 2.985***	− 1.708***
Cerebrovascular diseases (%)	− 4.522***	− 7.189***	− 6.153***	− 4.534***
Heart failure (%)	− 4.071***	− 8.889***	− 11.01***	− 12.63***
Osteoarticular diseases (%)	− 6.603***	− 12.35***	− 15.25***	− 16.30***
Depression (%)	− 2.272***	− 3.895***	− 4.795***	− 6.316***
Chronic kidney disease (%)	− 1.889***	− 4.544***	− 4.895***	− 5.005***
Cancer (%)	− 0.543***	− 0.905***	− 0.625***	− 0.216**
Sleep apnea (%)	− 1.285***	− 3.931***	− 7.023***	− 11.36***
PCOS (%)	1.098***	0.712***	0.571***	0.276*
*No. of comorbidities (%)*				
0	31.64***	50.36***	54.26***	56.59***
1–3	− 30.17***	− 46.47***	− 49.31***	− 50.92***
> 3	− 1.467***	− 3.890***	− 4.946***	− 5.670***
Glycemic status (%)				
Normoglycemia (NGT)	12.8***	27.6***	33.5***	37.5***
Impaired fasting glucose (IFG)	− 32***	− 5.5***	− 4.4***	− 3***
Diabetes mellitus (DM)	− 9.6***	− 22.1***	− 29.1***	− 34.4***

Hip and knee osteoarthrosis

Esophagus, stomach, intestine, colon, rectum, liver, gallbladder, pancreas

Polycystic ovary syndrome

**p* < 0.05; ***p* < 0.01; ****p* < 0.001

^3^ and

**Table 4:** T-test on mean differences for values reported in Table 2

Comorbidities	Normal weight		Overweight		Obesity class 1		Obesity class 2		Obesity class 3	
	NGT vs IFG	NGT vs DM	NGT vs IFG	NGT vs DM	NGT vs IFG	NGT vs DM	NGT vs IFG	NGT vs DM	NGT vs IFG	NGT vs DM
Hypertension	− 32.5***	− 52.3***	− 24.5***	− 32.5***	− 18.8***	− 26.2***	− 19.6***	− 24.3***	− 19.9***	− 24.06***
Dyslipidemia	− 26.4***	− 29.05***	− 18.8***	− 15.4***	− 16.4***	− 15.6***	− 14.8***	− 15.6***	− 8.9***	− 16.07***
Coronary heart disease	− 2.4***	− 6.6***	− 3.3***	− 5.5***	− 3.1***	− 5.1***	− 1.7***	− 4.7***	− 0.2***	− 2.5***
Cerebrovascular disease	− 7.3***	− 16.3***	− 6.9***	− 10.3***	− 5.8***	− 9.9***	− 4.1***	− 8.01***	− 2.02***	− 5.06***
Heart failure	− 3.6***	− 10.9***	− 4.5***	− 8.9***	− 4.6***	− 10.1***	− 5.5***	− 11.3***	− 7.1***	− 12.05***
Osteoarticular diseases	− 5.2***	− 9.8***	− 5.4***	− 6.3***	− 4.3***	− 6.2***	− 6.1***	− 7.3***	− 6.7***	− 9.5***
Depression	− 4.04	− 6.3***	− 1.2***	− 2.7***	− 1.2***	− 3.06	− 1.5***	− 3.08***	0.8***	− 3.4***
Chronic kidney disease	− 2.6***	− 8.6***	− 2.3***	− 6.05***	− 2.3***	− 7.02	− 2.3***	− 7.6***	− 0.03***	− 6.3***
Cancer	− 1.9***	− 3.3***	− 1.01***	− 1.9***	− 0.5***	− 1.2***	− 0.7***	− 1.5***	0.04***	− 0.9***
Sleep apnea	− 0.9***	− 0.6***	− 1.5***	− 0.9***	− 2.3***	− 1.6***	− 4.06***	− 3.3***	− 2.4***	− 4.8***
PCOS	0.8***	0.8***	0.2***	0.07***	0.5***	0.5***	0.4***	0.5***	0.4***	0.4***
*No. of comorbidities*										
0	36.8***	56.6***	25.8***	32.4***	18.4***	24.3***	18.2***	22.2***	16.4***	21.8***
1–3	− 35.4***	− 50.8***	− 23.8***	− 27.7***	− 16***	− 18.2***	− 15.4***	− 15.5***	− 13.9***	− 22.7***
> 3	− 1.4***	− 5.7***	− 2***	− 4.7***	− 2.4***	− 6.1***	− 2.8***	− 6.7***	− 2.5***	− 6.1***

Hip and knee osteoarthrosis

Esophagus, stomach, intestine, colon, rectum, liver, gallbladder, pancreas

Polycystic ovary syndrome

*NGT* normoglycemia, *IFG* impaired fasting glucose, *DM* diabetes mellitus

**p* < 0.05; ***p* < 0.01; ****p* < 0.001

^4^.

## Appendix D

Distribution of participating General Practitioners by Italian regions (N = 800).
